# The Simple Ailments of Horses: Their Nature and Treatment

**Published:** 1882-07

**Authors:** 


### REVIEWS.

---

The Simple Ailments of Horses: Their Nature and Treatment. By W. F. Landon. Paris and New York: Cassell, Petter, Galpin & Co. 1882.

Veterinarians and owners of horses will find some interest and no little profit in this small octavo of about 200 pages. The style is simple and attractive, while there is no attempt at any scientific treatment of the subject. Instead of any classification of the different diseases of the animal, the author arranges them alphabetically, with a descriptiom ot each disease under the name, and also proper remedies to be used. We would call attention particularly to an interesting chapter on "How to Move a Sick Horse."

